# Personalization of Cancer Treatment: Exploring the Role of Chronotherapy in Immune Checkpoint Inhibitor Efficacy

**DOI:** 10.3390/cancers17050732

**Published:** 2025-02-21

**Authors:** Rosalyn M. Fey, Avery Billo, Terri Clister, Khanh L. Doan, Elizabeth G. Berry, Deanne C. Tibbitts, Rajan P. Kulkarni

**Affiliations:** 1 Department of Dermatology, Oregon Health & Science University, Portland, OR 97239, USA; feyr@ohsu.edu (R.M.F.);; 2 Division of Oncological Sciences, Oregon Health & Science University, Portland, OR 97239, USA; 3 Knight Cancer Institute, Oregon Health & Science University, Portland, OR 97239, USA; 4 Cancer Early Detection Advanced Research Center (CEDAR), Portland, OR 97239, USA; 5 Operative Care Division, U.S. Department of Veterans Affairs Portland Health Care System, Portland, OR 97239, USA

**Keywords:** chronotherapy, immune checkpoint inhibitor, cancer, circadian rhythms, time-of-day administration, immune-related adverse events, side effects, sex differences, infusion timing, immunotherapy

## Abstract

In the era of precision medicine, mounting evidence suggests that the time of therapy administration, or chronotherapy, has a great impact on treatment outcomes. Chronotherapy involves planning treatment timing by considering circadian rhythms, which are 24 h oscillations in behavior and physiology driven by synchronized molecular clocks throughout the body. The value of chronotherapy in cancer treatment is currently under investigation, notably in the effects of treatment timing on efficacy and side effects. Immune checkpoint inhibitor (ICI) therapy is a promising cancer treatment. However, many patients still experience disease progression or need to stop the therapy early due to side effects. There is accumulating evidence that the time of day at which ICI therapy is administered can have a substantial effect on ICI efficacy. Thus, it is important to investigate the intersections of circadian rhythms, chronotherapy, and ICI efficacy. In this review, we provide a brief overview of circadian rhythms in the context of immunity and cancer. Additionally, we outline current applications of chronotherapy for cancer treatment. We synthesize the 29 studies conducted to date that examine the impact of time-of-day administration on the efficacy of ICI therapy, its associated side effects, and sex differences in both efficacy and side effects. We also discuss potential mechanisms underlying these observed results. Finally, we highlight the challenges in this area and future directions for research, including the potential for a chronotherapeutic personalized medicine approach that tailors the time of ICI administration to individual patients’ circadian rhythms.

## Simple Summary

Immune checkpoint inhibitor (ICI) therapy for cancer is promising but often results in adverse side effects and is not consistently effective. Emerging research suggests that the efficacy of ICIs may be improved by the application of chronotherapy—administering treatment at a specific time of day. In this article, we review the literature investigating time-of-day-dependent outcomes of ICI administration. We also explore potential mechanisms underlying this trend and consider challenges and future directions in this nascent field of study.

## 1. Introduction

Chronotherapy tailors treatment timing according to circadian rhythms—24 h oscillations in behavior and physiology regulated by molecular clocks. This strategy has gained attention in cancer treatment applications for its potential to enhance treatment efficacy and minimize side effects. One area of interest is the timing of immune checkpoint inhibitor (ICI) therapy, which is a highly effective cancer treatment for some patients. However, tumor resistance or side effect development can limit the broader applicability of ICI therapy. Primary resistance occurs when tumors fail to respond from the onset of treatment, while secondary resistance emerges following a period of tumor regression, ultimately leading to disease progression despite continued treatment [1]. The development of ICI-associated side effects, termed immune-related adverse events (irAEs), further complicates treatment and may necessitate therapy cessation. Emerging evidence suggests that the time of day at which ICI therapy is administered can significantly influence its efficacy and potentially associated toxicities [2]. This review provides an overview of circadian rhythms in the immune system and cancer, explores the current applications of chronotherapy in cancer treatment, and synthesizes research on time-of-day effects on ICI efficacy and toxicity. Additionally, we discuss the literature investigating potential mechanisms underlying the time-of-day-dependent immune response to ICI therapy, and we highlight key challenges and future directions in this evolving field, including the possibility of a chronotherapeutic precision medicine approach that emphasizes the importance of tailoring ICI therapy to an individual’s circadian profile.

## 2. Methods

**Figure 1:**
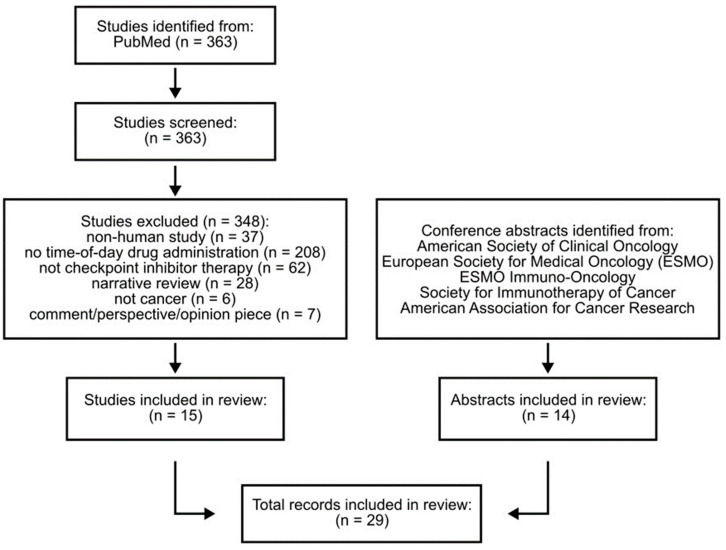
Flow diagram depicting the selection process used to identify articles examining time-of-day effects of ICI administration on ICI efficacy and toxicity.

This review identified published data that evaluated the impact of time-of-day administration of immune checkpoint inhibitors. Studies were included if they were published between 2011 and 2024, described a study conducted in human subjects with cancer, and reported any outcome based on time-of-day administration of a checkpoint inhibitor (alone or combined with another cancer therapy). PubMed was searched for published articles (search performed on 26 August 2024); narrative reviews returned by our search were hand-searched for primary references and abstracts. Databases of relevant conferences were also searched for published abstracts (searches performed on 20–22 August 2024). Abstracts from the following annual meetings were searched: the American Society of Clinical Oncology, European Society for Medical Oncology (ESMO), ESMO Immuno-Oncology, Society for Immunotherapy of Cancer, and American Association for Cancer Research. We used the following search terms to identify articles and abstracts: immune checkpoint inhibitor/blockade, immunotherapy, ipilimumab, pembrolizumab, nivolumab, atezolizumab, avelumab, durvalumab, cemiplimab, tremelimumab, dostarlimab, toripalimab, retifanlimab, cancer/neoplasms, chronotherapy/drug chronotherapy, circadian rhythm, time-of-day, infusion timing, and time-dependent/timing-dependent. Study identification, screening, and selection are depicted in Figure 1.

## 3. What Are Circadian Rhythms?

Circadian rhythm is an evolutionarily conserved time-keeping system that regulates a variety of processes (including sleep–wake cycles, eating–fasting cycles, and activity–rest cycles) allowing organisms to make adaptations to predictable environmental changes that occur around the day–night cycle [3]. The primary circadian clock is located in the suprachiasmatic nucleus (SCN) in the hypothalamus and tracks the environmental illumination cycle with light input signals derived from the retina. The circadian mechanism utilizes multiple negative and positive feedback loops to ensure stable and precise function. BMAL1, a core component of the molecular circadian clock, is regulated by retinoid-related orphan receptors (RORs), which activate its expression, and REV-ERBs, which repress it [4]. The transcription factors CLOCK and BMAL1 heterodimerize in the cytoplasm and enter the nucleus to activate the transcription of Period and Cryptochrome genes by binding to the E-box motif in their promoter regions. Once translated, PER and CRY proteins inhibit the activity of the CLOCK–BMAL1 complex, creating another cycle of activation and repression. Disruption to the circadian rhythms due to environmental factors (e.g., jet lag, shift work) contributes to the development of multiple diseases including cardiovascular disease, neurodegenerative disorders, sleep disorders, and cancer [3]. In addition, various cancer hallmark pathways, tumor suppressors, and oncogenes are under the control of the core circadian clock. Recent reviews further explored the role of the circadian clock in tumor suppression [5,6].

## 4. What Is Immune Checkpoint Inhibitor Therapy?

Immune checkpoint inhibitor (ICI) therapy uses monoclonal antibodies to boost the anti-cancer immune response by targeting the major immune checkpoint molecules: programmed cell death protein 1 (PD-1) and its ligand PD-L1 and cytotoxic T-lymphocyte-associated protein 4 (CTLA-4) [7]. PD-1 is expressed on the surface of activated T cells, B cells, and NK cells, and its ligand PD-L1 is present on various types of tumor cells and participates in the inhibition of activated T cells. CTLA-4 is present on the surface of CD4+ and CD8+ lymphocytes and binds to T cell costimulatory factors on the surface of antigen-presenting cells (APCs). Since 2011, multiple ICIs have been developed and approved by the FDA for a variety of cancer types. Although effective in treating cancer, these therapies can generate autoimmunity in the form of immune-related adverse events (irAEs), which comprise over 70 different pathologies impacting nearly every organ system [8]. The severity of symptoms varies, and up to 90% of patients develop mild side effects (grade 1–2). More severe grades are less common but can be fatal [9]. For grades 1 and 2 irAEs, symptoms can be managed without stopping ICI treatment, whereas grades 3 or higher irAEs necessitate ICI discontinuation and the initiation of corticosteroids, though the field is moving towards more targeted steroid-sparing treatments [9].

## 5. Introduction to Cancer Chronotherapy

The concept of linking therapy to circadian rhythm (chronotherapy) to improve the outcomes of patients with cancer has been discussed and tested since the late 1960s [10]. Chronotherapy can refer to either changing a patient’s circadian rhythm (as through lifestyle changes or drugs targeting circadian mechanisms [11]) or changing the timing of drug administration [12]. Due to the ubiquity of the circadian rhythm in bodily processes, changing the timing of drug administration can impact efficacy through the drug’s mechanism of action and metabolism of the drug itself, both of which can be under circadian influence [13,14,15]. Most clinical trials for cancer chronotherapy have focused on changing the timing of cancer therapy.

A very early example comes from a 1970s case study of a young patient with a malignant ovarian endodermal sinus tumor that also had spillage into the peritoneal cavity [16]. After surgical removal of the tumor, timing for the subsequent combination chemotherapy (cyclophosphamide, actinomycin, and vincristine) was systematically changed across her first four treatments. Extensive blood characterization and quality-of-life assessment during the treatments were used to determine the treatment time that was best tolerated by this patient. After finishing her therapy using that timing, the patient lived for over 30 years in remission when the expected 2-year survival was originally 10% for her malignancy. Retrospective studies and prospective clinical trials since then have further tested the influence of timing on outcomes for radiation, cytotoxic chemotherapy, and, more recently, ICI therapy.

Radiation is a targeted treatment that induces DNA damage in order to destroy cancer cells. Because DNA damage repair mechanisms are strongly influenced by circadian pathways, timing radiation therapy to occur when DNA repair mechanisms are less active seems mechanistically promising to improve treatment efficacy [17]. However, multiple reviews that evaluated clinical trials studying the impact of radiation timing on cancer treatment outcome and toxicity found inconclusive or contradictory results [12,18,19,20,21,22,23]. Despite unclear efficacy changes with chrono-radiation therapy, one common theme in these clinical trials is improved toxicity depending on the timing of radiation treatment.

Similar to radiation chronotherapy, clinical trials measuring the efficacy of cytotoxic chemotherapy-based chronotherapy had contradictory results depending on the specifics of the study [12,19,20,23,24,25]. In a recent systematic review that analyzed 18 randomized clinical trials (involving 2544 patients), only three studies demonstrated a statistically significant difference in efficacy due to the timing of the chemotherapy dose [26]. However, a majority of the studies (n = 11, 61%) showed a significant decrease in the severity, onset, or duration of side effects. Collectively, the most consistent result of clinical trials for both radiation and chemotherapy across cancers is that chronotherapy has a larger impact on adverse side effects compared to the efficacy of the treatment itself.

## 6. Effect of Time of Day of ICI Administration on Efficacy

**Figure 2:**
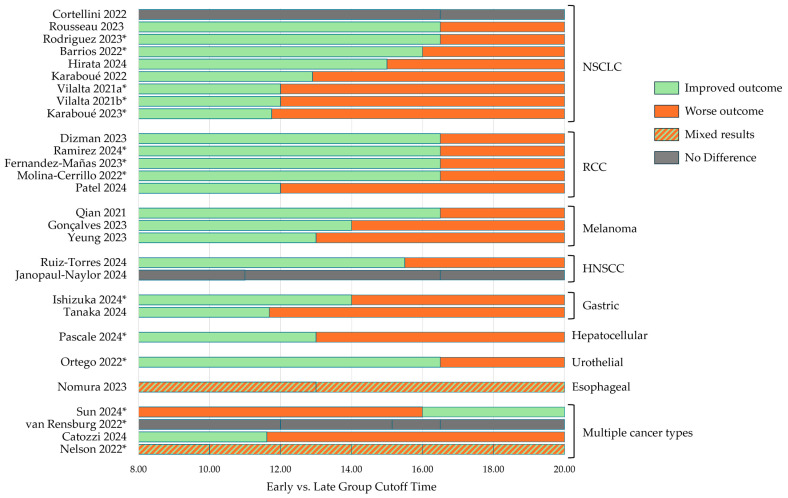
Graphical summary of efficacy outcomes from analyzing time-of-day administration of ICIs. Vertical bars within each row indicate time-of-day boundaries chosen by the study; time windows on either side of the time-of-day boundary are shaded according to improved (green) or worse (orange) outcomes. Studies with mixed results (hatched pattern) or no difference (gray) are shaded uniformly across time windows. Studies are grouped by cancer type evaluated: non-small cell lung cancer (NSCLC), renal cell carcinoma (RCC), melanoma, head and neck squamous cell carcinoma (HNSCC), gastric cancer, hepatocellular carcinoma, urothelial cancer, esophageal cancer, or if the study included multiple types of cancer. Conference abstracts are marked with an asterisk (*). The meta-analysis by Landre (2024) is excluded from this graph, but all the studies it included are listed [27,28,29,30,31,32,33,34,35,36,37,38,39,40,41,42,43,44,45,46,48,49,50,51,52,53,54,55].

Unlike the body of the literature on chrono-radiotherapy and chrono-chemotherapy, multiple studies showed that the efficacy of ICI therapy is dependent on the time of administration. To date, there have been no prospective, randomized trials published investigating this connection; therefore, the evidence that we reviewed here was abstracted from retrospective, secondary, and meta-analyses. Many study design factors, including the outcomes investigated and the dosing cutoff times that stratify individual patients into early or late treatment groups, varied among studies (Table 1 and Figure 2). However, the emerging literature largely suggests that dosing earlier in the day results in more favorable patient outcomes.

**Table 1:** Summary of overall and sex-specific differences in efficacy outcomes in studies reporting time-of-day effects of ICI administration.

Study	Cancer	ICI	N	TOD Cutoff (24-h Clock Format)	Grouping Strategy	TOD of Better Efficacy Outcomes	Efficacy Outcomes	Significant Effects of Time of Day on Efficacy Outcomes (*p* ≤ 0.05)	% Female	Sex-Specific Differences in TOD Response? (*p* ≤ 0.05)
Catozzi 2024 [27]	metastatic or unresectable locally advanced solid tumors	Atezo, Durva, Nivo, Pembro	361	11:37	AM group: median infusion time (>50% of infusions) before cutoff; PM group: median infusion time (>50% of infusions) after cutoff	AM	OS, ORR	longer OS and better ORR in AM group	39%	OS, ORR: AM better for females, no difference for males; amplitude of timing impact greater for females
Cortellini 2022 [28]	metastatic NSCLC	Pembro	262 total, 180 matched	16:30	AM group: < 20% of infusions after cutoff; PM group: >=20% after cutoff	No difference	OS, PFS	no difference in OS or PFS	50% overall, 50% matched	NR
Dizman 2023 [29]	metastatic RCC	Nivo, Ipi/Nivo	135	16:30	AM group: <20% of infusions after cutoff; PM group: >=20% after cutoff	AM	OS, ORR, time to treatment failure (TTF)	shorter TTF in multivariate analysis in PM group; no difference in ORR or OS; shorter TTF and OS in PM group for alternate infusion percentage cutoffs of 25% and 30%	30%	NR
Gonçalves 2023 [30]	stage IV melanoma	Nivo, Pembro, Ipi/Nivo	73	14:00	AM group: <75% of infusions after cutoff; PM group >=75% after cutoff	AM	OS, PFS	shorter OS in PM group, no difference in PFS	38%	OS: AM better for females, no difference for males; PFS: no difference for females or males
Hirata 2024 [31]	locally advanced NSCLC	Durva	82	15:00	AM group: <20% of infusions after cutoff; PM group: >=20% after cutoff	AM	OS, PFS	shorter PFS in PM group, no difference in OS	18%	NR
Janopaul-Naylor 2024 [32]	metastatic HNSCC	Nivo	62	11:00 and 16:30	AM group: first infusion before morning cutoff; mid-day group: first infusion between morning and evening cutoffs; PM group: first infusion after evening cutoff	No difference	OS, PFS, ORR	no difference between the three groups in OS, PFS, ORR	NR	NR
Karaboué 2022 [33]	metastatic NSCLC	Nivo	95	12:54	AM group: majority of infusions before cutoff; PM group: majority of infusions after cutoff	AM	OS, PFS, ORR, DCR	higher OS and PFS in AM group; larger ORR and DCR in AM group	17%	NR
Landré 2024 [34]	metastatic solid tumors	anti-PD-1, anti-PD-L1, or dual anti PD-1/CTLA-4	1663	12:00, 12:54, 13:00, 14:00, 16:00, or 16:30	AM groups: 25%, 50%, 75%, 80%, >=1 infusions before cutoff time	AM	OS, PFS, ORR	meta-analysis of OS, PFS longer in AM groups; ORR higher in AM group in 4 of 6 studies	33%	NR
Nomura 2023 [35]	metastatic or recurrent esophageal squamous cell carcinoma	Nivo	62	13:00	early-first: first infusion before cutoff, late-first: first infusion after cutoff; early-3M: >=50% of infusions in the first 3 months before cutoff, late-3M: <50% before cutoff; early-all: >=50% of all infusions before cutoff, late-all: <50% before cutoff	AM	OS, PFS, ORR, DCR	longer PFS and higher ORR in AM group for -first and -3M comparisons, no difference for -all comparisons; OS longer in AM group for -first comparison, no difference for -3M and -all comparisons; no difference in DCR for any comparison	19%	first dose analysis: PFS: AM better for males, no difference for females; OS: no difference for males or females; ORR: NR
Patel 2024 [36]	stage IV RCC	Nivo, Pembro, Ipi/Nivo	201	12:00	AM group: >=20% of infusions before cutoff; PM group: <20% before cutoff	AM	OS, PFS, ORR	longer OS, PFS and higher ORR in AM group	27%	OS: no difference for males or females; PFS, ORR: NR
Qian 2021 [37]	stage IV melanoma	Ipi, Nivo, Pembro, Ipi/Nivo	299 total, 146 matched	16:30	AM group: <20% of infusions after cutoff; PM group: >=20% after cutoff	AM	matched: OS; unmatched: PFS, CRR	matched: shorter OS in PM group; unmatched: lower PFS in PM group, no difference in CRR	34% overall; 37% matched	OS: AM better for females, no difference for males; PFS, CRR NR
Rousseau 2023 [38]	advanced NSCLC	Atezo, Nivo, Pembro	180	16:30	AM group: <20% of infusions after cutoff; PM group: >=20% after cutoff	AM	OS, PFS	shorter PFS in PM group in multivariate analysis, but no difference after adding “total #” of infusions received" to model; no difference in OS	38%	NR
Ruiz-Torres 2024 [39]	recurrent, advanced, or metastatic HNSCC	Durva, Ipi, Nivo, Pembro, any dual ICI	113 total, 98 matched	15:00	AM group: <20% of infusions after cutoff; PM group: >=20% after cutoff	AM	OS, PFS	overall: shorter PFS in PM group, no difference in OS; matched: shorter PFS in PM group, each additional 20% of infusions in PM associated with shorter OS	31% overall, 30% matched	NR
Tanaka 2024 [40]	stage IV gastric cancer	Nivo	58	11:41	AM group: median administration time of each patient’s infusions before cutoff; PM group: median administration time after cutoff	AM	OS, PFS, ORR, DCR	longer OS, PFS and higher ORR, DCR in the AM group	17%	NR
Yeung 2023 [41]	advanced unresectable or metastatic melanoma	anti-PD-1, anti-PD-L1, or dual ICIs	121	13:00	AM group: >=1 of the first 4 infusions before cutoff; PM group: all first 4 infusions at/after cutoff	AM	OS, PFS, ORR	shorter OS and PFS in PM group; no difference in ORR	37%	NR
Conference Abstracts										
Arroyave Ramirez 2024 [42]	metastatic RCC	Ipi/Nivo	127	16:30	AM group: <20% of infusions after cutoff; PM group: >=20% after cutoff	AM	OS, ORR	longer OS and higher ORR in AM group	30%	NR
Barrios 2022 [43]	advanced NSCLC	Atezo, Nivo, Pembro	508	16:00	AM group: <=20% of infusions after cutoff; PM group: >20% after cutoff	AM	median time to treatment discontinuation of ICI	increased risk of treatment discontinuation in PM group	34%	NR
Fernandez-Mañas 2023 [44]	metastatic RCC	anti-PD-1 or anti-PD-L1	104 total, 56 analyzed	16:30	AM group: <20% or 50% of infusions after cutoff; PM group: >=20% or 50% after cutoff	AM	OS, ORR, time on treatment (TOT), time to next treatment (TNT)	20%: shorter OS and TOT in PM group; 50%: shorter TOT, TNT, and higher frequency of progressive disease in PM group	26% overall; 23% second- and later-line group	NR
Ishizuka 2024 [45]	metastatic gastric cancer	Nivo	248	14:00	AM group: >=70% of infusions before cutoff, PM group: <70% before cutoff	AM	OS, PFS, ORR, DCR	longer OS, PFS and higher ORR, DCR in AM group	NR	NR
Karaboué 2023 [46]	stage IV NSCLC	Pembro	97	11:45	AM group: 2–4 of initial 4 infusions before cutoff; PM group: 0–1 of initial 4 infusions before cutoff	AM	OS, PFS	longer OS in AM group; no difference in PFS	30%	NR
Molina-Cerrillo 2022 [47]	metastatic RCC	Pembro, Ipi/Nivo	61	16:30	AM group: <=20% of infusions after cutoff; PM group: >20% after cutoff	AM	OS, PFS	longer PFS in AM group; OS data immature	NR	NR
Nelson 2022 [48]	advanced solid tumors	anti-PD-1, anti-PD-L1, or dual anti PD-1/CTLA-4	4441	every 2 h from 8:00 to 20:00, and ’overnight’ (20:00 to 8:00)	NR	Mixed	OS, PFS	overnight group had worse OS vs 10:00–16:00 group for lung, renal, breast cancers; overnight, 8:00–10:00, and 18:00–20:00 groups had worse OS vs 10:00–16:00 for melanoma; 8:00–10:00 group had worse OS vs 12:00–16:00 group for head & neck cancer; PFS similar	42%	NR
Ortego 2022 [49]	metastatic urothelial cancer	anti-PD-1 or anti-PD-L1	92	16:30	AM group: <20% of infusions after cutoff; PM group: >=20% after cutoff	AM	OS, PFS	longer OS and PFS in AM group	NR	NR
Pascale 2024 [50]	advanced hepatocellular carcinoma	Atezo	131	13:00	AM group: >=1 of the first two infusions before cutoff; PM group: first two infusions after cutoff	AM	OS	shorter OS in PM group	9%	NR
Rodriguez 2023 [51]	advanced or metastatic NSCLC	Pembro	276	16:30	AM group: first infusion before cutoff; PM group: first infusion after cutoff	AM	OS, PFS	shorter OS in PM group, no difference in PFS	NR	NR
Sun 2024 [52]	advanced cancer	Camre, Tisle	174 total; 109, Camre cohort; 65, Tisle cohort	16:00	AM group: <1 infusion after cutoff; PM group: >=1 infusion after cutoff	PM	PFS	longer PFS in PM group overall and for camrelizumab cohort; no difference in PFS for tislelizumab cohort	NR	NR
van Rensburg 2022 [53]	advanced solid tumors	Pembro	106	12:00, 15:06, 15:11, or 16:30	AM groups: first infusion before 12:00 or 15:06, >=50% of infusions before 12:00 or 15:11, <20% of infusions after 16:30; PM groups: first infusion after 12:00 or 15:06, >=50% of infusions after 12:00 or 5:11, >=20% of infusions after 16:30	No difference	OS, PFS	no difference in OS or PFS for any comparison	NR	NR
Vilalta 2021a [54]	NSCLC	anti-PD-1	197	12:00	AM group: >=1 of the first 4 infusions before cutoff; PM group: all 4 first infusions after cutoff	AM	OS, PFS, DCR	longer OS and PFS in AM group in multivariate analysis; no difference in DCR	28%	NR
Vilalta 2021b [55]	NSCLC	anti-PD-1	105	12:00	AM group: >=1 of the first 4 infusions before cutoff; PM group: all 4 first infusions after cutoff	AM	PFS, response rate at first radiologic evaluation	longer PFS in AM group; no difference in RR	27%	NR

Landré 2024: Note that not all proportions were applied to each time-of-day cutoff. Abbreviations: NSCLC—non-small-cell lung cancer, RCC—renal cell carcinoma, HNSCC—head and neck squamous cell carcinoma, Atezo—Atezolizumab, Durva—Durvalumab, Nivo—Nivolumab, Pembro—Pembrolizumab, Ipi—Ipilimumab, Camre—Camrelizumab, Tisle—Tislelizumab, TOD—time of day, OS—overall survival, ORR—objective response rate, PFS—progression-free survival, DCR—disease control rate, CRR—complete response rate, NR—not reported.

The first paper on this topic was published in 2021. Qian et al. showed that, among patients receiving ICIs for stage IV melanoma, those receiving 20% or more of their infusions after 16:30 had shorter overall survival (OS) in both unmatched and propensity score-matched analyses [37]. Importantly, the 16:30 cutoff was chosen to represent the onset of “evening” based on previous studies of circadian immune function and to accommodate clinic hours. The reported results remained robust in multivariate analyses (accounting for factors such as age, receipt of corticosteroids within one month of any infusion, and receipt of radiotherapy), suggesting that earlier infusion times may result in more favorable patient outcomes.

Fourteen more peer-reviewed research articles and 23 conference abstracts investigating the effect of ICI infusion timing on patient outcomes have been published since this initial study (Figure 1). Of these conference abstracts, nine were eventually published as full-length articles. Of the 28 studies (research articles and conference abstracts) published since Qian et al., 23 (82%) showed that earlier ICI administration resulted in better efficacy in at least one measured outcome (e.g., overall survival, progression-free survival) [27,29,30,31,33,34,35,36,38,39,40,41,42,43,44,45,46,47,49,50,51,54,55]. Three studies (11%) reported no difference in measured outcomes [28,32,53], one study (4%) reported mixed results depending on the measured outcome [48], and one study (4%) reported better outcomes when ICIs were administered later in the day [52]. Each of these studies is further detailed in Table 1 and Figure 2. While these studies may be grouped by overall effect, there are multiple study design factors that affect the interpretation and comparison of these reports. Below, we detail several of the most important considerations and highlight the methods used to define patient groupings and time-of-day cutoff.

### 6.1. Outcomes

Measured outcomes differed from study to study. Almost all studies investigated overall survival (OS) (27 out of 29, 93%), except for two conference abstracts [43,52]. Barrios et al. focused on time to treatment discontinuation [43], while Sun et al. examined only progression-free survival (PFS) [52]. Importantly, Sun et al. are the only group so far to report more favorable outcomes in patients who received ICI later in the day (one or more infusions administered after 16:00) [52]. The majority of studies also reported PFS (23, 79%). Just under half reported either objective (ORR) or complete response rate (CRR) (12, 41%). Other measured outcomes included disease control rate (DCR) (two studies, 7%) and some variation in time and treatment interaction (time to treatment failure or discontinuation, time on treatment, or time to next treatment; three studies, 10%).

### 6.2. Allocation into Early and Late Administration Groups

In the included studies, patients were stratified into early and late administration groups based on time of day. One of the most important differences in these studies was the decision to assign patients to these groups based on either the percentage of total infusions or initial infusions received before a time-of-day cutoff. Several early studies demonstrated the importance of the timing of initial infusions for patient outcomes. In a 2022 study by Karaboué et al., landmark analyses revealed that after two months of treatment, the late administration group already had significantly worse OS and PFS, suggesting that the timing of initial ICI treatments is important for efficacy [33]. Later in 2022, Cortellini et al. found that the total number of infusions received was the strongest determinant of patient outcomes and demonstrated that adjusting for this factor reduced OS and PFS from 47.1 and 19.7 months to 29.0 and 11.8 months, respectively, in patients receiving <20% of infusions in the evening [28]. Since then, multiple studies specifically considered outcomes by initial infusions (10 of 29, 31%). Of these, three reported no difference between earlier and later administration, with initial infusions defined as the first dose only [29,32,53]. Seven studies found more favorable results when initial infusions were administered earlier in the day. Of these, two also considered only the first infusion and found more favorable outcomes in the early administration group [35,51]. Other studies defined the early administration of initial infusions as receiving at least one of the first four [41,54,55] or one of the first two [50] doses before the time-of-day cutoff or receiving two to four of the first four doses before the cutoff [46]. For the studies stratifying patients into early or late administration groups based on the proportion of total infusions received before the time-of-day cutoff, various percentages were considered (Table 1).

### 6.3. Time-of-Day Cutoff

The cutoff time to determine early or late in the reviewed studies varied widely (Figure 2). As previously described [34], these may have fallen into two broad categories: those chosen for biological reasons and those chosen for practical reasons, such as statistical or clinical constraints. Of the 29 studies, 19 (66%) reported the reasoning behind the choice of time-of-day cutoff. Two (of 19, 11%) studies reported consulting the body of the literature on circadian immunity to choose their cutoffs. As a rationale for this choice, Qian et al. cited pre-clinical and clinical studies of vaccination timing and circadian lymphocyte variation [37], while Janopaul-Naylor et al. cited studies of CD8 T cell diurnal concentrations [32]. Most studies (11 of 19, 58%) reported using the previous literature (i.e., the study by Qian et al. [37]) to choose their cutoff. Two of these (of 11, 18%) adjusted the cutoff to accommodate the clinic’s closing hours and/or statistical considerations, in both instances shifting the cutoff to 15:00 (versus 16:30) [31,39]. Seven of these 19 studies (37%) reported choosing time-of-day cutoffs based on other practical considerations. The most common method involved calculating the median time of all patient infusion times (5 of 7, 71%). Most of these studies, therefore, had seemingly arbitrary cutoff times (11:41 [40], 11:45 [46], 12:54 [33], 15:06 and 15:11 [53]), with the exception of Patel et al., who rounded to the nearest hour (12:00) [36]. One study [27] used a predictiveness curve method to objectively determine the optimal cutoff for separating patients into early and late groups based on overall survival, resulting in a cutoff time of 11:37. Nomura et al. chose as the cutoff the midpoint of clinic opening hours (13:00) [35].

Ten studies (of 29 reviewed, 34%) did not report the reasoning behind the choice of time-of-day cutoff. We note that eight of these ten were conference abstracts, which are subject to space constraints. Of these ten studies, nine (90%) investigated only one time-of-day cutoff. The other [48] had an unusual grouping strategy: patients were grouped into two-hour windows from 8:00 to 20:00 (i.e., 8:00–10:00, 10:00–12:00, 12:00–14:00, etc.) and one overnight cohort from 20:00 to 8:00. The authors reported that patients with head and neck cancer had shorter OS when receiving infusions between 8:00 and 10:00 compared to 12:00–16:00. Patients with lung, renal, and breast cancers who received overnight infusions had significantly shorter OS compared to patients receiving infusions from 10:00–16:00; patients with melanoma had shorter OS when receiving overnight infusions but also when infusions were administered between 8:00 and 10:00 or 18:00 and 20:00 (compared to 10:00 and 16:00). We note that the shorter OS observed for overnight treatment windows may be confounded by a higher likelihood of overnight infusions for hospitalized (and potentially sicker) patients compared to outpatients.

### 6.4. Type of Cancer and Type of ICI

The majority of studies (24, 83%) focused on a single cancer type (Table 1). Of these 24, 9 (38%) were in non-small cell lung cancer (NSCLC), 5 in renal cell carcinoma (21%), 3 in melanoma (13%), and 2 each (8%) in head and neck squamous cell carcinoma (HNSCC) and gastric cancer. There was one instance (4%) each of esophageal cancer, urothelial cancer, and hepatocellular carcinoma. The majority of these studies (22 of 24, 92%) found improved outcomes with early ICI administration. One study of patients with HNSCC found no difference among early, mid, and late administrations of ICI on OS, PFS, or objective response rate [32]. Similarly, Cortellini et al. found no difference between early and late administrations on OS or PFS in patients with NSCLC [28]. It is interesting to note that, altogether, there were 13 studies that included patients with lung cancer (4 that examined multiple cancer types, 9 that studied only NSCLC). Of these, three (23%) found that early administration did not improve patient outcomes [28,48,52].

The remaining five studies analyzed multiple cancer types. Interestingly, three of these studies (all conference abstracts) reported results that do not corroborate the findings of Qian et al. Nelson et al. reported mixed results in patients with solid tumors (including melanoma, breast, colon, head and neck, liver, lung, and renal cancers) [48]. Many of these patients (31%) had non-small cell lung cancer. Van Rensburg et al. reported no difference between early and late ICI administrations in patients with advanced solid tumors (including melanoma, ovarian, head and neck squamous cell, and triple negative breast cancers) [53]. Sun et al. reported that later administration of ICI is more favorable for patients with advanced solid tumors (including esophageal and lung cancer) [52]. The other two studies that examined multiple cancer types were a meta-analysis [34] and a single center retrospective study [27]. The patients included in the meta-analysis by Landré et al. had esophageal carcinoma, melanoma, non-small-cell lung cancer, renal cell carcinoma, or urothelial cancer [34], while those in the study by Catozzi et al. had melanoma or breast, colorectal, head and neck, pancreatic, urinary, or non-small cell lung cancers [27]. Both reported more favorable outcomes with earlier ICI administration.

Of the 29 studies that we reviewed, 22 (76%) specified the ICI that patients received, while the remaining 7 reported only the target molecule (Table 2). Based on the varied results of time-of-day ICI administration reported thus far, we anticipate that the nuances of specific ICIs may become an important consideration and we encourage authors to include this information in future studies.

### 6.5. Sex-Specific Differences in ICI Efficacy by Time-of-Day Administration

**Figure 3:**
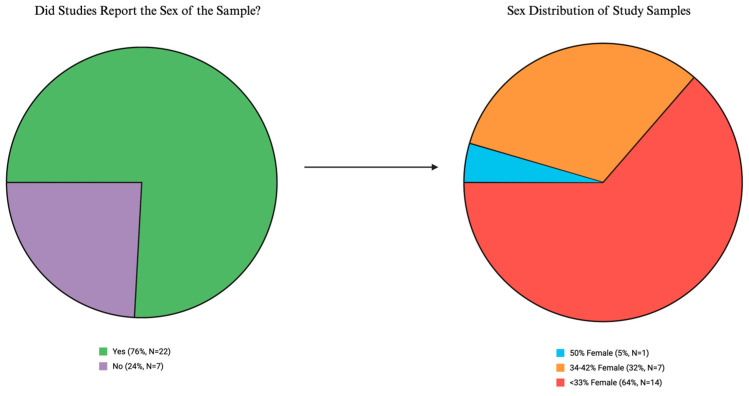
Pie chart summary of the proportion of studies reporting on the sex of study participants (n = 29) and the proportion of females in the sample for each of the studies we reviewed.

Some ICI studies suggest that efficacy may differ by patient sex, but overall results are mixed [56,57,58,59]. To synthesize the evidence on potential sex differences in ICI efficacy outcomes based on time-of-day administration, we examined each study for the proportion of females in the sample and whether studies reported on sex differences in efficacy outcomes. Most studies (76%) reported the sex of the sample, and, of those, female patients comprised less than 50% of the sample in all but one of the studies reviewed (Figure 3). 

Reporting of sex-specific differences in efficacy outcomes was rare. Only five studies (of 22, 23%) analyzed outcomes by patient sex [27,30,35,36,37]. Of these five studies, three reported better OS [27,30,37] for females in the early administration group and no difference in OS by time-of-day administration for males. Two studies reported no sex differences in OS [35,36]. Interestingly, Catozzi et al. applied a novel sinusoidal Cox regression model to assess the impact of time-of-day administration on OS and predict the best and worst time of day for ICI infusion [27]. Notably, they found that the amplitude of the timing effect was higher for females than for males. For PFS, one study reported better PFS for males in the early administration group (based on first dose administration) and no difference for females [35], and one study reported no sex differences in PFS [30]. Finally, one study reported better ORR for females in the early administration group and no difference in ORR by time-of-day administration for males [27].

Overall, the under-representation of female patients in these studies and limited reporting on sex-specific differences in efficacy are major gaps in the existing literature on ICI time-of-day administration and hinder evaluation of whether chronotherapy may differentially benefit male or female patients. Unfortunately, this trend is reflective of a landscape of female under-representation in clinical trials [60] and deficiencies in sex-based outcomes reporting in oncology [61] despite NIH policy to consider sex as a biological variable [62] and development of the SAGER guidelines [63] to improve sex-based reporting in manuscripts.

Although each of these studies has unavoidable biases due to their retrospective natures, the accumulating evidence suggests that the time of day at which ICI is administered influences patient outcomes in many scenarios. In contrast to studies of chemotherapy and radiotherapy, which report significant differences in time-of-day-dependent toxicity but not treatment outcomes, current studies of patients receiving ICI suggest that earlier administration results in better treatment efficacy. We note that there is a lack of consistency in these results; therefore, prospective, randomized, and rigorous studies are essential to understand the conditions under which timing ICI administration will be reproducibly beneficial.

## 7. Effect of Time of Day of ICI Administration on Toxicity

While we found 29 studies examining efficacy outcomes, fewer studies examined relationships between ICI time-of-day administration and toxicities (Table 3). Of the studies that reported toxicity data (12 of 29, 41%), 10 studies [27,30,31,32,33,37,39,40,41,53] reported on toxicity by time-of-day administration; the remaining 2 studies [35,52] reported overall toxicities but did not analyze by time-of-day administration. Toxicity-related outcomes by time-of-day administration included the prevalence of toxicities overall or by grade (7 of 12, 58%), prevalence of specific toxicities (2 of 12, 17%), ICI discontinuation due to toxicity (3 of 12, 25%), and ICI change due to toxicity (1 of 12, 8%).

Results were mixed on whether time-of-day administration affected ICI toxicity. Of the seven studies reporting on toxicity frequency, two studies found a higher prevalence of overall toxicities in the early administration group [27,41] while one did not [40]. Two studies showed a higher prevalence of higher-grade toxicities in the early group [27,30], while two did not [32,53]. Of the two studies examining specific types of toxicities, Karaboué et al. found that the most common toxicities among patients with NSCLC were fatigue and skin toxicities, with grade 3–4 fatigue occurring more frequently in the late administration group (15% late vs. 6% early) and grade 2–3 skin toxicities occurring more frequently in the early group (32% early vs. 13% late); other toxicities were infrequent and similar between early and late groups [33]. Gonçalves 2023 found no difference in toxicity types between early and late administration groups [30]. No study found differences in ICI discontinuation or ICI change due to toxicity [31,37,39].

As ICI toxicities were associated with efficacy outcomes in previous studies [64,65,66], we examined whether efficacy and toxicity are linked by time-of-day administration. Four studies that found a difference in toxicities by time-of-day administration also found a difference in a measure of efficacy, with higher toxicity rates and increased efficacy occurring together in early administration groups. Catozzi et al. found better OS, better ORR, and more frequent and higher-grade toxicities in the early group in a study of patients with mixed cancer types [27]. Yeung et al. found longer OS and PFS and a higher prevalence of toxicities in the early administration group in patients with advanced melanoma [41]. Karaboué et al. found longer OS and PFS and a more frequent occurrence of skin toxicities in the early group in patients with NSCLC [33]. Gonçalves et al. found that grade 3–4 toxicities were only reported in the early group, coupled with longer OS, in patients with stage IV melanoma [30]. Conversely, four studies showed improvement in efficacy outcomes in early groups without finding a difference in toxicities by time-of-day administration [31,37,39,40]. However, three of these four studies used ICI discontinuation as the toxicity-related outcome, which may not be a sensitive measure for potential relationships between efficacy and toxicity as ICI discontinuation is driven by higher-grade toxicities.

Finally, only one study stratified toxicity-related outcomes by patient sex. Catozzi et al. found a greater number and higher grade of toxicities in the early administration group for females but found no difference in these outcomes by time-of-day administration for males. The same study also reported better OS and ORR in females in the early group [27].

Overall, toxicity-related outcome reporting is inconsistent, and, when reported, the choice of outcome is also inconsistent. The standardization of toxicity reporting will help fully elucidate whether time-of-day administration affects the frequency, severity, or type of irAEs among patients treated with ICIs. Additionally, given documented sex differences in drug responses [67], toxicity data stratified by patient sex are necessary for optimizing the clinical benefit of chronotherapy as applied to ICIs.

## 8. Elucidating the Circadian Mechanism of ICI Efficacy and Toxicity

**Figure 4:**
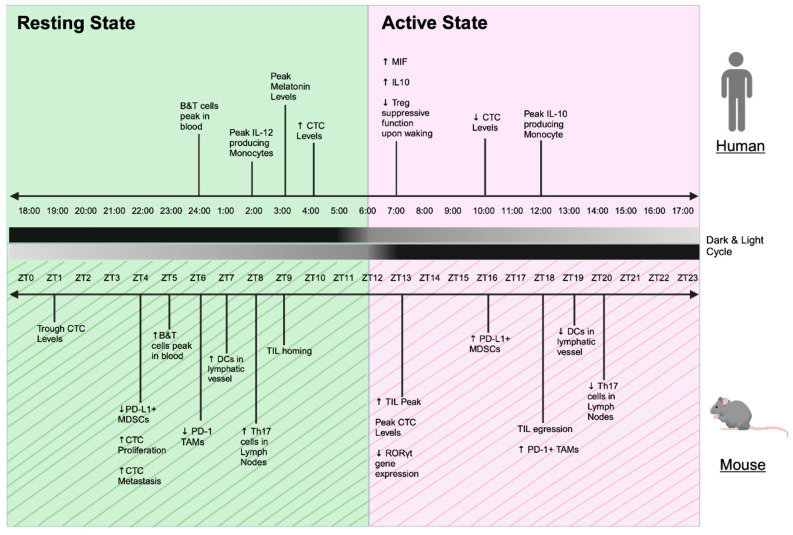
The alignment of the circadian activity/resting phase between mouse and human with their associated immune response as potential mechanisms for the improved ICI response and reduced irAEs’ development during early morning. ZT0 and ZT12 correspond to lights on and lights off in mice, respectively, and vice versa in human. Up arrows indicate upregulation or increased levels, and down arrows indicate downregulation or decreased levels. Abbreviations: CTC—circulating tumor cell, MDSCs—myeloid-derived suppressor cells, TAMs—tumor-associated macrophages, DCs—dendritic cells, TIL—tumor-infiltrating lymphocyte, Th17—T helper type 17, IL—interleukin, MIF—macrophage migration inhibitory factor, Treg—regulatory T cell.

Over the past decade, our understanding of the mechanisms of immune checkpoint inhibitors has greatly improved. ICI therapies restore anti-tumor immunity by blocking the regulatory signals that inhibit T cell activity [68]. However, the mechanisms of action surrounding other aspects of immune checkpoint inhibition, such as the development of irAEs and resistance, are not fully understood. Therefore, it is imperative that we identify new avenues to maximize the efficacy and minimize the toxicity of ICI therapy. Specifically, studies of the mechanisms of chronotherapy and circadian rhythm that influence the time-of-day efficacy of ICIs are limited to mostly pre-clinical murine studies. Although mice are nocturnal, they are valuable model organisms for immunological mechanistic investigations. To more easily translate between murine (nocturnal) and human (diurnal) physiology, Zeitgeber Time (ZT) is used in circadian studies. In the context of a 24 h light/dark cycle (12 h of light and 12 h of dark), ZT0 corresponds to the time at which lights turn on and ZT12 corresponds to the time at which lights turn off (Figure 4). Here, we highlight pre-clinical (murine) and clinical (human) studies to extrapolate a circadian link to ICI as potential mechanisms of action for chronotherapy. To do so, we discuss the role of PD-1 and its circadian control in different immune cell types and pathways, including tumor-associated macrophages (TAMs), myeloid-derived suppressor cells (MDSCs), macrophage migration inhibitory factor (MIF) and its cognate receptor CD74, circulating tumor cells (CTCs), and T cells/Tregs. We also discuss the migration of immune cells under ICI treatment and potential circadian-influenced mechanisms for irAEs induced by ICI treatment.

### 8.1. Linking the Sleep–Wake Cycle to Cancer

The sleep–wake cycle, a fundamental aspect of circadian rhythm, is crucial for regulating immune system function and response to external stimuli, influencing both the efficacy and toxicity of ICIs through these mechanisms. During normal sleep–wake cycles, T-helper 1 (Th1) cell activity, driven by IFN-γ-producing cells, is promoted at the start of the nighttime rest period, while T-helper 2 (Th2) activity, driven by IL-4-producing cells, dominates just before waking or during the later part of the rest period [69]. Sleep deprivation leads to an imbalance between Th1 and Th2 cell-derived cytokines, resulting in their excessive production and subsequent immune disturbances, which can lead to chronic inflammation and tissue damage [69]. Disruption in the sleep–wake cycle also causes the loss of the rhythmic temporal variations in IL-12 and IL-10 [69]. IL-12 is involved in naive T cell differentiation into Th1 cells, promotes IFN-y expression, and regulates T cell and natural killer cell responses [70]. IL-10 is an anti-inflammatory cytokine that downregulates Th1 cytokines, enhances B cell survival, and can help promote Th2 differentiation [71]. During sleep, monocytes producing IL-12 are increased and monocytes producing IL-10 are decreased; thus, nocturnal sleep represents the shift between IL-10 (Th2) activity and IL-12 (Th1) activity [69,72]. This circadian rhythm is disrupted during sleep deprivation, and the temporal variations between IL-12 and IL-10 monocytes are lost, causing immune system disfunction. Other noted effects of a sleep–wake cycle imbalance include decreased numbers and the weak lytic activity of NK cells, a decrease in CD8+ T cells, and an amplification of abnormal inflammatory responses including the activation of soluble adhesions molecule (sICAM)-modulated NF-kB inflammatory signaling and the cellular expression of inflammatory markers IL-6, TNF-a, and C-reactive protein (CRP) [3]. Therefore, sleep deprivation may lead to immune system changes that increase the risk of cancer.

Various cytokines such as TNF-a, TGF-b, IL-10, IL-1b, and IL-6 are involved in the crosstalk between cancer initiation/progression and sleep–wake cycles. IL-1b is pro-tumoral, having been shown to be upregulated in many solid tumors, promoting cancer progression [73]. This cytokine was also identified in the brain as a key mediator that affects rhythmical behavior, and sleep deprivation was found to stimulate the expression of IL-1b in the brain. IL-6 is upregulated and abundant in almost all types of tumors, which promotes tumorigenesis and facilitates the repair and induction of countersignaling pathways to protect cancer cells [74]. IL-6 is expressed at low levels during the day and peaks during sleep. Sleep deprivation results in the elevation of plasma levels of circulating IL-6. Both IL-6 and IL-1b illustrate how the sleep–wake cycle is connected to cytokines that play a role in cancer initiation/progression and how the disruption of the cycle is potentially a risk factor for tumorigenesis.

Endocrine factors like growth hormone, prolactin, thyroid hormone, cortisol, and gonadal steroids are governed by circadian rhythms and fluctuate throughout the day. Shift workers are at a higher risk of obesity and weight gain (increased body mass index) compared to dayworkers, potentially due to a mismatch in circadian rhythms from sleep deprivation [75]. Sleep deprivation triggers the decrease in levels of the appetite-restraining adipokine leptin and an increase in the levels of the appetite-stimulating peptide ghrelin, resulting in artificially inflated feelings of hunger and appetite. The hormonal imbalances associated with sleep deprivation lead to increased hunger, reduced satiety, higher cortisol levels, and impaired insulin sensitivity, which contribute to weight gain and can induce hyperplasia, a risk factor for cancer [75].

Disruption of the sleep–wake cycle can increase cancer risk by impairing immune system functionality and affecting pathways critical to ICI efficacy. Insufficient sleep and irregular sleep patterns, such as those experienced by shift workers, are known to influence cancer initiation, progression, and treatment [76]. For instance, sleep dysregulation leads to elevated levels of pro-inflammatory cytokines, such as TNF-α. Notably, TNF inhibition was shown to enhance ICI anti-tumor efficacy while reducing irAEs [77]. Sleep deprivation, a significant disruptor of the sleep–wake cycle, is a known cancer risk factor and can also influence ICI efficacy and the occurrence of immune-related adverse events (irAEs).

### 8.2. The Circadian Regulation of PD-1 and PD-L1

ICI drugs function by blocking checkpoint molecule interactions to maintain the functional role of immune cells, especially T cells. It is important to highlight that PD-1 suppresses the functionality of both cytotoxic and regulatory T cells [78]. Patients with high levels of PD-1-expressing T cells have better clinical response on ICIs than those who have low levels of PD-1-expressing T cells [78]. Additionally, anti-PD-1 increases PD-1 CD8+ TCR clonality after treatment, which is correlated with a positive clinical outcome in patients [79,80]. Regulatory T cells (Tregs) are a subset of CD4+ T helper cells that exhibit an immunosuppressive function and are capable of attenuating TCR signaling in effector T cells [81]. Notably, ICIs induce both a specific effector memory T cell subset that is associated with positive outcome and a separate Treg subset that is associated with toxicity [80]. In a sleep study with healthy volunteers, the number of peripheral blood Tregs peaked and remained steady during sleeping hours (19:00–7:00) and decreased shortly after waking up, with a nadir around 11:00. Congruently, Treg suppressive activity on CD4+ T cells was high during sleep, with peak suppression at 2:00 and lowest at 7:00, upon waking [82]. Early morning infusion of ICIs would thus be during a time when Tregs (and their suppressive function) within the peripheral blood are low, enabling a greater T cell-mediated immune response and TCR production. This concept also introduces a sleep-dependent mechanism (versus time-of-day) as a potential explanation for ICI response and toxicity.

Macrophages are a type of innate immune cell that mature from monocytes upon migration to tissue [83]. Upon infiltrating a tumor, macrophages play an important role in eliminating cancer cells by producing chitotriosidase/chitinases, proteases, nitric oxide, and hydrogen peroxide [84]. However, cancer cells can foster immune escape from macrophages by expressing the anti-phagocytosis regulator CD47 [85] or by polarizing macrophages into an anti-inflammatory/immunosuppressive M2 phenotype, which are commonly known as tumor-associated macrophages (TAMs) [86,87]. TAMs are associated with tumor progression and poor prognosis in many cancer treatment modalities [88,89,90]. In the context of ICIs, TAMs can express PD-1 and, upon PD-1 inhibition, anti-tumor immunity is improved in a macrophage-dependent manner [91]. In mice, the number of PD-1-expressing TAMs oscillates diurnally [92]. Administration of an anti-PD-1/PD-L1 agent at ZT18 (during active state), when PD-1-expressing TAMs peak, results in significantly increased phagocytic activity of TAMs and tumor growth suppression compared to ZT6 (during resting state at the TAM PD-1-expression nadir) [92]. Whether this circadian behavior and treatment response by TAMs translate to humans needs further investigation. We can infer that the TAMs’ peak during the activity phase at ZT18 in mice can correspond to the middle of the activity phase of humans at late morning/noon (Figure 4). Furthermore, low levels of monocytes in human peripheral blood during the early morning [93] could suggest an increase in the migration and maturation of macrophage in tissue, increasing the overall number of PD-1-expressing macrophages and thus the effect of anti-PD-1 therapy during the early morning.

Myeloid-derived suppressor cells (MDSCs) are a subset of myeloid cells, often neutrophil- and monocyte-derived, that exhibit potent immunosuppressive activity [94]. MDSCs are a strong contributor to the poor clinical outcomes seen in cancer [95,96]. PD-L1-expressing MDSCs peak in tissues at ZT16 (active phase) in WT mice compared to ZT4 (resting phase). Disruption in the circadian clock gene *Bmal1* in intestinal epithelial cells in a mouse model of colorectal cancer abrogated this difference between ZT4 and ZT16 in the intestinal microenvironment, demonstrating that the fluctuation of MDSCs (and their PD-L1 expression) is circadian rhythm-dependent [97]. In the same mouse model, ICI administration at ZT16, when PD-L1+ MDSCs are at their highest, significantly reduces disease burden compared to ICI administration at ZT4 [97]. This response corroborates the time-dependent increase in ICI efficacy during the active phase in other mouse studies. In many cancer types where tumors overexpress PD-L1 and PD-L2, anti-PD-1 therapy significantly improves clinical outcomes compared to patients whose tumors have low PD-L1 and PD-L2 expression [98,99,100]. Altogether, administration timing of ICIs can influence their efficacy based on the oscillatory expression of immune checkpoint molecules on immune cells and tumors, which often peak early during the active phase.

Likewise, the macrophage migration inhibitory factor (MIF) is a diurnally regulated cytokine that peaks during early to late morning [101,102] and upregulates PD-L1 expression in melanoma cells [103]. MIF also plays a role in MDSC differentiation and T cell immunosuppression [104]. In our previous review, we highlighted the role of MIF and its cognate receptor CD74 as potential biomarkers for ICI therapy response and irAE development [105]. ICI administration in the morning may counteract the immunosuppressive functionality of MIF at its peak to reinvigorate the anti-tumor response and improve the clinical response of ICI [106,107].

Circulating tumor cells (CTCs) can express PD-L1, which inhibits the anti-tumor response in circulation and contributes to poor clinical outcomes in multiple cancers [108,109,110,111]. Accordingly, patients with high PD-L1+ CTCs had a higher response rate from ICIs than those who did not [112,113]. This established a correlative mechanism by which the CTCs can affect the efficacy of ICIs. An early study attempted to identify a diurnal oscillation of CTCs and found no significant difference in the level of CTCs at 8:00 versus 20:00 in patients with metastatic breast cancer [114]. A more recent and extensive study evaluated the CTC level at 4:00 (resting phase) and 10:00 (activity phase) in patients with breast cancer and found that the CTC level during the resting phase was higher than during the activity phase [115]. However, the peak level of CTCs was not determined and was postulated to be within the early morning (6:00 to 9:00). Interestingly, the same publication found CTCs exhibited greater metastatic potential during the resting phase in mice. This could be attributed to the observed higher cell proliferation and cell division occurring in the body (and, reasonably, tumors) during sleep [116]. Unfortunately, it is difficult to implement treatment with ICIs during natural rest/sleep times to combat this phenomenon. However, this process was found to be malleable in mice: the proliferative and metastatic potentials of tumors and CTCs were instead greater during the active phase upon insulin administration (upregulating cell growth and division) [115]. In another mouse study, CTC counts reached a peak at ZT13 (activity phase) and nadir at ZT1 (resting phase) [117]. ZT13 in mice (one hour into the activity phase) corresponded closely to early morning in humans (Figure 4). Thus, ICI infusion in the morning could allow the immune system to eliminate CTCs and reduce the risk of metastasis, providing a potential mechanism for improving OS and PFS.

### 8.3. Diurnal Migration Within the Immune System

Lymph nodes (LN) are important structures for immune activation and surveillance [118,119]. Murine studies demonstrated that lymphocyte egress and homing to and from the lymph nodes are under diurnal circadian control [120,121]. In mice, B cells and T cells peak in the blood at ZT5 (the middle of the resting phase), then gradually decrease to a trough at ZT13 (the start of the activity phase), and remain low throughout ZT13–20 (during the activity phase) [120]. In the mouse model of experimental autoimmune encephalomyelitis (EAE), disease severity (as a function of immune surveillance and activation) worsened when inoculation happened at ZT8 compared to ZT20, and Th17 cells, the T cell subset responsible for driving the EAE autoimmune response [122,123], were found to be significantly higher in the LN at ZT8 during LN homing compared to ZT20 during LN egress [121]. Thus, lymphocytes within the LN played a greater role than within the peripheral blood in the immune response and induction of EAE. Likewise, B and T cells in the LN were significantly elevated and remained high throughout the activity phase [120]. Additionally, in a mouse melanoma model, tumor size was significantly larger when tumor cells were inoculated at ZT21 (near the end of the activity phase) compared to ZT9–ZT13 (near the star8t of the activity phase) [124]. The number of leukocytes, including naive and activated CD4 and CD8 T cells, significantly increased 24 h later in the draining LN in the cohort inoculated at ZT9, demonstrating the time-of-day influence on leukocyte proliferation as a mechanism for anti-tumor immunity. A higher infiltrate of dendritic cells (DCs) was also found in the lymphatic vessels (LVs) at ZT7 than ZT19 [125] and exhibited greater costimulatory factors in T cell activation at ZT9 [124], when they traveled from LVs to LNs.

In addition to lymphocytes peaking in the LN at ZT13, leukocyte infiltration of tumors also peaked at ZT13 in mice [126]. The adoptive transfer of T cells at ZT13 resulted in a higher infiltration in tumor and better tumor control compared to ZT1, and anti-PD1 administration at ZT13 also enhanced tumor control [126]. Interestingly, the cell number of leukocyte infiltration was approximately equal before (ZT9) and after (ZT18), peaking at ZT13 [126]. This raises the question of the functional role of individual tumor-infiltrating lymphocytes (TILs) during homing versus egress and their functional association to ICIs. The administration of ICIs at ZT9 in this study may have acted to further militarize leukocytes homing to tumors, leading to better tumor control at ZT13, but this remains to be elucidated. Together, these data indicate the relevance of circadian timing in relation to the location, proliferation, and activation of leukocytes during disease inoculation and ICI administration.

### 8.4. IL-10

Interleukin 10 (IL-10) is a pleiotropic cytokine and is produced by many immune cells, including T cells, B cells, DCs, and NK cells [127,128]. In its pro-inflammatory role, IL-10 is secreted by both Th1 and Th2 cells, while, when it is anti-inflammatory, it is secreted by regulatory T cells (Tregs) [129]. IL-10 is diurnally influenced by the clock gene REV-ERB, a key modulator of the circadian rhythm in mammals [130,131]. In human peripheral blood cytokine studies, high baseline IL-10 levels before ICIs were found to be prognostic of disease progression but also of irAEs [132,133,134] and lower serum and a reduction in IL-10 levels during ICIs were associated with better ICI efficacy [135,136]. IL-10 is known to exhibit an autoregulatory downregulation of MHCII expression in monocytes and limit the production of pro-inflammatory cytokines [130,137]. In addition, IL-10-producing B cells are capable of suppressing T cell proliferation and inducing Treg cells [138]. In fact, pro-inflammatory cytokines peak during nighttime, whereas IL-10 peaks during the day [139]. Thus, the administration of ICIs during the daytime (activity phase) versus nighttime (resting phase) may also play a secondary role in overriding both the anti-inflammatory and pro-inflammatory functionality of IL-10 to improve ICI efficacy while possibly decreasing irAE development. However, studies also highlighted the pleiotropic and contradictory role of IL-10 in the context of irAEs, with an increase in IL-10 during ICI being associated with a reduced risk of irAEs [140], or reduction in IL-10 being associated with an increased risk of irAEs [136], varying over multiple cycles of treatment.

Relatedly, a study on the irAE development based on the calendar season found the highest incidence of irAEs in patients who started ICI during the winter season [141]. The increase in specific irAEs throughout the season was attributed to the extrinsic factors unrelated to ICI treatment, such as the patient lifestyle, environmental condition, and infectious disease that can prime the immune system and influence irAE development [141,142]. Chronotherapy is therefore not limited to time of day and may need to be expanded to include seasonal and environmental fluctuations as well.

### 8.5. Th17 Cells and irAEs

Th17 cells are a subset of CD4+ T helper (Th) cells characterized by their production of the cytokine interleukin-17 (IL-17) [143]. In studies of immune-related (ir) toxicities of ICIs, tissues with irColitis and irRash showed an upregulation of CD4+ T cells with IL17A expression, and the subsequent administration of secukinumab (anti-IL-17A) in these patients abrogated their respective irAEs [144,145,146]. Likewise, a higher starting baseline of circulating IL-17 levels significantly increased the risk of developing severe (grade 3+) irColitis/diarrhea [132]. Studies showed that Th17 proliferation and IL-17 secretion are regulated by the circadian clock, in particular, the orphan nuclear receptor RORgt [147]. Th17 cells were found to be higher at ZT16 (activity phase) than at ZT4 (resting phase) in the small intestine in mice [148]. The trafficking of Th17 cells into the small intestine was facilitated by the CCR6/CCL20 axis [149], which was also controlled by the circadian clock [150]. CCL20 mRNA levels in tissues were found to be higher at ZT8 than at ZT20 [148], suggesting the accumulation of CCL20 expression in the small intestine at ZT8 later influenced the tissue homing of Th17 cells at ZT16. The single-cell analysis of immune-related tissues in ICI patients revealed greater infiltration of Th17 cells compared to healthy tissues [144,151,152]. A subset of CD8 T cells in irColitis also shifted toward a Th17 phenotype, as inferred from the increased expression of IL17A, IL26, IL23R, and BATF [151]. An analysis of intestinal group 3 innate lymphoid cells (ILC3s), the innate counterpart of Th17 cells, found RORgt expression was low at ZT13, although there were no differences in the number of ILC3s [153]. Antagonizing RORgt as a transcription factor repressed the Th17-mediated pro-inflammatory function [154]. In summary, Th17 cells promote autoimmune disorders, including irAEs in ICI, and Th17-mediated inflammation is diurnally regulated. Therefore, administering ICI in the early morning could reduce the development of irAEs, potentially due to the lower presence of Th17 cells during this time. Detecting such an effect in patients would depend on identifying the optimal infusion timing window and selecting appropriately granular clinical measures of toxicity (e.g., tissue-specific), as existing retrospective studies relied on heterogeneous timing cutoffs and toxicity-related outcomes.

### 8.6. Melatonin and the Immune System

Melatonin is a hormone released from the pineal gland and functions to synchronize the circadian rhythm and sleep. Melatonin was also shown to modulate both the human immune system and the tumor microenvironment, exerting a pro-immunogenic effect on T cells, Tregs, IL-10, and TAM and MDSC polarization as well as contributing to the regulatory modulation of Th17 cells (extensively reviewed by Mu and Najafi) [155]. The effects of melatonin on the immune system provide another potential mechanism linking the circadian rhythm and sleep to ICI efficacy and toxicity.

In humans, endogenous melatonin levels increase shortly after the start of the dark cycle (18:00), peak during sleep (between 2:00 and 4:00), and gradually return to baseline at awakening (~8:00) [156]. However, the endogenous secretion and exogenous intake of melatonin are different, as the over-the-counter supplement increases melatonin levels as much as 10–100 times higher than the peak level during sleep [156]. Endogenous melatonin regulates sleep, but exogenous melatonin may be used to enhance immune system function. A preliminary clinical study [157] showed that patients with solid cancers had better disease control on a high-dose melatonin/anti-PD-1 combination compared to anti-PD-1 alone. A recent murine study showed that a melatonin/anti-PD-L1 combination reduced tumor growth and metastasis compared to anti-PD-L1 alone after radiofrequency ablation [158]. Melatonin was also shown to inhibit PD-L1 expression in tumor cells [158,159,160,161]. In summary, melatonin is a regulator of sleep and serves as a bridge between the circadian rhythm and the immune system, and these studies support the further investigation of exogenous melatonin as a potential treatment modality to enhance the anti-tumor efficacy of ICIs.

## 9. Future Perspectives: Elucidating the Circadian Mechanism of ICI Efficacy and Toxicity

### 9.1. Investigating the Paradox of Immune Therapies

Although drugs with short half-lives are generally considered to be ideal for chronotherapeutic applications, the studies reviewed here suggest that even modalities with longer half-lives (on the order of weeks) may be effective chronotherapeutics. This may seem counterintuitive, as drugs with longer half-lives often obscure circadian effects [162]; however, additional factors play a role, including the time it takes the drug to reach steady-state concentrations in the body and the circadian rhythmicity of target cells or molecules.

Depending on the drug, it takes 4 to 18 weeks for ICIs to reach steady-state concentrations [163]; therefore, there is a crucial pre-steady-state window in which the time of day of administration may be leveraged to maximize effectiveness and improve patient outcomes. In addition, multiple studies reviewed here focused on the time-of-day effect of initial infusions. Specifically, Nomura et al. found that administering initial infusions in the morning resulted in improved patient survival and response rates but that the effect of time-of-day treatment was lost when considering all courses [35]. This provides additional evidence for the special consideration of the time-of-day administration of the first ICI infusions.

Another potential explanation of this paradoxical circadian effect is the rhythmicity of target cells. It was suggested that T cells are more susceptible to activation or stimulation early in the day; therefore, administering ICI therapy when T cells are most ready to be activated may increase efficacy regardless of the half-life of the drug or whether steady state has been reached [164]. While current pharmacokinetic and pharmacodynamic models cannot explain this phenomenon, these ideas provide potential avenues of exploration for future studies.

### 9.2. Assessing Personal Circadian Rhythm

Our understanding of the circadian rhythm and its impact on the immune system, tumor cells, and drug pharmacology has increased dramatically in the past decade [67]. In the instance of ICIs, as circadian control of the immune system is better understood, it becomes possible to design clinical trials of ICIs that incorporate chronobiology principles, including a patient’s personal chronobiology. Differences in circadian rhythm are common between sexes [67] and between chronotypes (i.e., morning vs. evening person). For example, females exhibit a greater number of rhythmically expressed genes across multiple tissues [165] and a higher amplitude of rhythmic plasma melatonin levels [166] than males. Disrupted sleep/wake cycles also impact personal chronobiology. For instance, inflammatory cytokines were more highly expressed in night-shift workers [167], and exposure to bright light during normal sleeping hours caused a phase-shift in circadian gene expression in the PBMCs of healthy volunteers [168]. These differences may have an important impact on treatment efficacy in chronotherapy for cancer.

Although the time-of-day effect of ICI therapy has not yet been established, there may be opportunities in the future to apply personal chronobiology principles to its administration. Randomized controlled chronotherapy trials including measurements of personal molecular circadian mechanisms were published for other cancer treatment modalities. For example, in a trial [169] evaluating radiotherapy timing (morning or evening) on breast cancer outcomes, it was found that morning treatment is associated with increased late-effect toxicity. However, that correlation was much more significant if patients were stratified by polymorphisms in specific circadian rhythm genes (PER3 and NOCT) [169]. Another trial of 166 patients with glioblastoma investigating the chemotherapy drug temozolomide (TMZ) found a significant difference in efficacy when treatment was given in the morning vs. the evening [170]. The increase in overall survival was driven by patients (N = 56) with methylated (i.e., silenced) promoter regions for MGMT (O-6-Methylguanine-DNA Methyltransferase). MGMT is the protein responsible for repairing TMZ-induced DNA damage, and its expression oscillates throughout the day. The patients who had MGMT expression suppressed at the time of treatment responded better to treatment. These examples show how individual chronobiology can play a critical role in therapy outcomes.

As this field expands, the opportunity will arise to include the evaluation and consideration of patients’ individual circadian rhythms. The current gold standard for identifying an internal circadian phase is dim light melatonin onset (DLMO), a proxy for the phase of the master circadian clock in the SCN [171,172]. The measurement of DLMO requires the individual to remain in clinic for up to 24 h while their melatonin levels are monitored via repeated blood or saliva sampling in a dim-light setting. For various reasons, this is impractical for patients with cancer, and cancer clinics are not set up to carry out these specialized protocols.

Fortunately, various alternatives are being developed. Several recent reviews describe efforts in this field [67,171,173,174]; therefore, we will only briefly highlight these methods. Personal circadian rhythm may be evaluated by wearable devices that take the continuous measurement of parameters such as core or surface body temperature, heart rate, activity, and light exposure [175,176,177]. These have a much lower patient burden compared to DLMO assessment because the devices can be worn at home. Another strategy for evaluating individual circadian time is to infer the circadian phase from a sample taken at a single point in time (snapshot methods). This is advantageous because it can be performed once in the clinic during a regularly scheduled visit. A variety of methods have been developed, including those using one to two samples of easily accessible tissues, fluids, or cell types, such as whole blood or blood-isolated monocytes, hair follicles, oral mucosa, or skin [173,178,179,180], and those applying machine learning methods to identify the molecular chronotype of a tissue sample [181]. Population-based methods combine a traditional around-the-clock sampling scheme with snapshot methods. A small number of individuals are monitored over the course of 24 h, and the remaining individuals are sampled at a single point in time. The single timepoint data are then modeled/matched to the around-the-clock data [182,183].

Although these methods are promising and address some of the practical difficulties of implementing DLMO in a cancer clinic setting, both wearable devices and snapshot methods need optimization to match the performance of DLMO, which remains the gold standard for evaluating personal circadian rhythms. In addition, many of these recently developed approaches have not been validated against DLMO or other gold standard methods, a prerequisite to using them to assess a personal circadian phase in the clinic for chronotherapeutic applications. However, in the absence of information on individual circadian rhythms, population-level data (e.g., sex differences in circadian biology) could be evaluated for their utility in personalizing treatment approaches.

### 9.3. Practical Considerations of Personalized Chronobiology

The understanding of the efficacy and toxicity mechanisms behind early ICI infusion and what timing should be considered “early” is important and remains to be elucidated before translation into the clinic. In this section, we highlighted the clinical challenges and logistics, particularly as they pertain to healthcare in the United States, for allocating patients on ICI to exclusively fit in the early/morning period for their infusion.

A typical treatment day for a patient on ICI consists of a lab visit, a consultation visit with a clinician, and a 3 h period for infusion, in that order. A clinician must address patient safety (through bloodwork), symptoms, and concerns before the patient can receive another cycle of treatment. Thus, planning to fit many patients within the “early morning” window can be difficult, especially if clinicians have a fixed clinic schedule. However, these requirements do not need to happen on the same day. It is possible for a patient to obtain their labs, have their provider visits, and then come back the subsequent day to start treatment if the timing happened to be too late during the day. Unfortunately, attending clinic multiple days in a row will be excessively burdensome for some patients, particularly those who need to travel farther for care, have caregiver responsibilities, and/or are not able to take time off work. Patients who are able to visit the clinic multiple days in a row may still face challenges that will increase the burden of care, including increased personal financial costs (such as the cost of staying overnight in a hotel) and increased travel to and from the clinic.

In addition, such a visit schedule could be a logistical challenge for the clinic and providers. Treatment in late afternoons or early evenings may often not be possible due to staff scheduling needs and the need for patient monitoring during infusions. However, some of the studies reviewed here demonstrated better efficacy outcomes when grouping patients based on the timing of initial infusions (first infusion, first four infusions, first 3 months) [35,41]; if future studies corroborate the importance of time-of-day administration for initial infusions more so than total infusions, then initial infusions could be prioritized for morning administration, lowering the burden on the clinic and patients.

Additionally, lifestyle and environmental factors can cause an individual and their chronobiology to shift and vary. For example, what constitutes “lights on” and “lights off” (and thus our activity and resting states) is the natural light–dark cycle from the sun (e.g., sunrise and sunset), which can vary depending on the yearly seasons. In addition, artificial lighting exposure can also have an influence on our circadian clock [184]. Control of one’s circadian rhythm, such as consistent sleep–wake cycles, may also be difficult due to a variety of factors (e.g., age, extent and variability of activity during the wake cycle, and sleep-related needs and symptoms due to disease). As a result, measuring and understanding the influence of chronotherapy is challenging due to the extreme personalized variations in patients.

However, as our knowledge of chronotherapy increases and the effect of early day infusion is clarified, we can foster a paradigm shift toward the inclusion of chronotherapy within personalized medicine. In the future, bloodwork performed before each ICI treatment cycle could be paired with circadian rhythm assessment to optimize delivery of therapy. The inclusion of chronotherapy in ICI treatment regimens will also require clinicians, hospital administrators, and insurance companies to collaborate to mitigate the increase in patient burden caused by more restrictive infusion timing or by increases in clinical assessments (such as circadian rhythm evaluation). Future studies may support the use of melatonin beyond the treatment of sleep difficulties to a tool to align the circadian rhythm to the optimal time window to receive ICI infusion (in addition to the immunogenic benefits of melatonin). Emphasizing sleep patterns and circadian rhythm management may also increase patients’ sense of agency over their care. Adjusting infusion schedules within the healthcare system to prioritize morning infusion timing for ICIs may be a particular challenge, but specific implementation hurdles would depend on the outcome of future research to understand the relative importance of initial infusion timing vs. overall infusions.

## 10. Conclusions

Chronotherapy for cancer treatment is a rapidly evolving area of research, with ICI treatment receiving recent attention. Despite some mixed results, the majority of the retrospective studies reviewed here suggest that earlier time-of-day administration of ICI therapy results in improved patient outcomes. The mechanism (or mechanisms) behind this phenomenon has not yet been elucidated; however, several hypotheses exist that could explain the reported results. Further experiments are needed to determine why early ICI administration appears more effective in some cases. Basic and translational science studies to uncover a mechanism will be complemented by further clinical studies on the effect of early ICI administration on patient outcomes that fully assess its potential benefit to patients. Various limitations affect the studies performed to date, especially their retrospective designs, highlighting the need for carefully designed prospective trials (e.g., NCT05549037) to determine the conditions under which ICI timing can maximize efficacy while minimizing toxicity. Relatedly, common difficulties emerge when comparing studies to discern the effect of chronotherapy. Difficulty splitting patients into distinct early and late groups due to scheduling issues or in the case of retrospective studies and choosing how to group patients whose treatments span the time groups make it difficult to standardize and directly compare studies. Similarly, infusion duration and dose intensity are known to impact the toxicity of chemotherapeutic agents [26]. Studies that do not control for these factors can confound the assessment of any potential benefit that exists for timing cancer treatments. Other limitations include the lack of systematized reporting of toxicities, sex-balanced enrollment, and stratification of results by patient sex. Future directions for this rapidly evolving field include the development of clinically practical methods to assess individual circadian rhythms and the investigation of how tailoring treatment timing to an individual patient’s chronobiology affects ICI efficacy and toxicity. We anticipate that future work in this area will propel the field of cancer chronotherapy forward for the benefit of patients.

## Figures and Tables

**Table 2:** Immune checkpoint inhibitors analyzed by time-of-day administration and their target molecules.

Immune Checkpoint Inhibitor	Target Molecule
Atezolizumab	PD-L1
Camrelizumab	PD-1
Durvalumab	PD-L1
Ipilimumab	CTLA-4
Nivolumab	PD-1
Pembrolizumab	PD-1
Tislelizumab	PD-1

**Table 3:** Summary of overall and sex-specific differences in toxicity outcomes in studies reporting time-of-day effects of ICI administration.

Study	Cancer	ICI	N	TOD Cutoff (24-h Clock Format)	Grouping Strategy	Toxicity, Grade, and % Reporting	Toxicity-Related Outcome	Significant Differences in Toxicity by Time of Day? (*p* ≤ 0.05)	Sex-Specific Differences in Toxicity by TOD? (*p* ≤ 0.05)
Catozzi 2024 [27]	metastatic or unresectable locally advanced solid tumors	Atezo, Durva, Nivo, Pembro	361	11:37	AM group: median infusion time (>50% of infusions) before cutoff; PM group: median infusion time (>50% of infusions) after cutoff	any toxicity, 40% (AM group = 49%, PM group = 34%); 1 toxicity, 27%, 2 toxcicites, 10%, 3 toxicities, 2%; any grade 1, 19%; any grade 2, 14%; any grade 3, 6%; any grade 4, 1%	frequency of toxicity by number and grade	greater number and higher grade of toxicities in AM group	greater number and higher grade of toxicities in AM group for females; no difference for males
Cortellini 2022 [28]	metastatic NSCLC	Pembro	262 total, 180 matched	16:30	AM group: <20% of infusions after cutoff; PM group: >=20% after cutoff	NR	NR	NR	NR
Dizman 2023 [29]	metastatic RCC	Nivo, Ipi/Nivo	135	16:30	AM group: <20% of infusions after cutoff; PM group: >=20% after cutoff	NR	NR	NR	NR
Gonçalves 2023 [30]	stage IV melanoma	Nivo, Pembro, Ipi/Nivo	73	14:00	AM group: <75% of infusions after cutoff; PM group >=75% after cutoff	(grade 1–4) fatigue, 37%; cutaneous, 33%; endocrine, 22%; renal insufficiency, 11%; hepatitis, 11%; pneumonitis, 4%; uveitis, 3%; encephalitis, 3%	frequency of grade 3–4 toxicity, toxicity by type	grade 3–4 toxicities were only reported in the AM group; no difference by toxicity type	NR
Hirata 2024 [31]	locally advanced NSCLC	Durva	82	15:00	AM group: <20% of infusions after cutoff; PM group: >=20% after cutoff	any toxicity, 13%; pneumonitis, 11%, endocrinopathies, 1%, peripheral neuropathy, 1%	ICI discontinuation	no difference	NR
Janopaul-Naylor 2024 [32]	metastatic HNSCC	Nivo	62	11:00 and 16:30	AM group: first infusion before morning cutoff; mid-day group: first infusion between morning and evening cutoffs; PM group: first infusion after evening cutoff	any grade 3–4 toxicity, 11% (AM group = 14%; mid-day group = 9%; PM group = 15%)	frequency of grade 3–4 toxicity	no difference	NR
Karaboué 2022 [33]	metastatic NSCLC	Nivo	95	12:54	AM group: majority of infusions before cutoff; PM group: majority of infusions after cutoff	(any grade) fatigue, 55%; skin toxicity, 26%; anorexia, 25%; anemia, 24%; nausea, 21%; hepatitis, 16%; renal failure, 16%; vomiting, 15%; hypothyroidism, 13%; muscle toxicity, 13%; diarrhea, 12% ; abdominal pain, 8%	frequency of toxicity by type	fatigue (grade 3/4) more frequent in PM group; skin toxicities (grade 2/3) more frequent in AM group; no difference for all other toxicities	NR
Landré 2024 [34]	metastatic solid tumors	anti-PD-1, anti-PD-L1, or dual anti PD-1/CTLA-4	1663	12:00, 12:54, 13:00, 14:00, 16:00, or 16:30	AM groups: 25%, 50%, 75%, 80%, >=1 infusions before cutoff time	NR	NR	NR	NR
Nomura 2023 [35]	metastatic or recurrent esophageal squamous cell carcinoma	Nivo	62	13:00	early-first: first infusion before cutoff, late-first: first infusion after cutoff; early-3M: >=50% of infusions in the first 3 months before cutoff, late-3M: <50% before cutoff; early-all: >=50% of all infusions before cutoff, late-all: <50% before cutoff	(grade 1–3) pruritis, 10%; hypothyroidism, 8%; pneumonitis, 5%; proteinuria, 2%; mucositis oral, 2%; colitis, 2%; diarrhea, 2%; hypopituitarism, 2%; rash maculopapular, 2%; bullous dermatitis, 2%; fatigue 2%; infusion-related reaction, 2%; fever, 2%	NR	NR	NR
Patel 2024 [36]	stage IV RCC	Nivo, Pembro, Ipi/Nivo	201	12:00	AM group: >=20% of infusions before cutoff; PM group: <20% before cutoff	NR	NR	NR	NR
Qian 2021 [37]	stage IV melanoma	Ipi, Nivo, Pembro, Ipi/Nivo	299 total, 146 matched	16:30	AM group: <20% of infusions after cutoff; PM group: >=20% after cutoff	colitis, 18%; hepatitis, 9%; hypophysitis, 5%; others <5%	ICI change, ICI discontinuation	no difference	NR
Rousseau 2023 [38]	advanced NSCLC	Atezo, Nivo, Pembro	180	16:30	AM group: <20% of infusions after cutoff; PM group: >=20% after cutoff	NR	NR	NR	NR
Ruiz-Torres 2024 [39]	recurrent, advanced, or metastatic HNSCC	Durva, Ipi, Nivo, Pembro, any dual ICI	113 total, 98 matched	15:00	AM group: <20% of infusions after cutoff; PM group: >=20% after cutoff	any toxicity that led to complete discontinuation of ICI, 6% (AM group = 4%, PM group = 10%)	ICI discontinuation	no difference	NR
Tanaka 2024 [40]	stage IV gastric cancer	Nivo	58	11:41	AM group: median administration time of each patient’s infusions before cutoff; PM group: median administration time after cutoff	any toxicity, 19% (AM group = 17%, PM group = 21%)	frequency of toxicity	no difference	NR
Yeung 2023 [41]	advanced unresectable or metastatic melanoma	anti-PD-1, anti-PD-L1, or dual ICIs	121	13:00	AM group: >=1 of the first 4 infusions before cutoff; PM group: all first 4 infusions at/after cutoff	any toxicity, 67% (AM group = 72%, PM group = 43%); any grade 1, 17%; any grade 2, 47%; any grade 3, 31%; any grade 4, 4%; any grade 5, 1%	frequency of toxicity	lower prevalence of toxicities in PM group; no difference by grade	NR
Conference Abstracts									
Arroyave Ramirez 2024 [42]	metastatic RCC	Ipi/Nivo	127	16:30	AM group: <20% of infusions after cutoff; PM group: >=20% after cutoff	NR	NR	NR	NR
Barrios 2022 [43]	advanced NSCLC	Atezo, Nivo, Pembro	508	16:00	AM group: <=20% of infusions after cutoff; PM group: >20% after cutoff	NR	NR	NR	NR
Fernandez-Mañas 2023 [44]	metastatic RCC	anti-PD-1 or anti-PD-L1	104 total, 56 analyzed	16:30	AM group: <20% or 50% of infusions after cutoff; PM group: >=20% or 50% after cutoff	NR	NR	NR	NR
Ishizuka 2024 [45]	metastatic gastric cancer	Nivo	248	14:00	AM group: >=70% of infusions before cutoff, PM group: <70% before cutoff	NR	NR	NR	NR
Karaboué 2023 [46]	stage IV NSCLC	Pembro	97	11:45	AM group: 2–4 of initial 4 infusions before cutoff; PM group: 0–1 of initial 4 infusions before cutoff	NR	NR	NR	NR
Molina-Cerrillo 2022 [47]	metastatic RCC	Pembro, Ipi/Nivo	61	16:30	AM group: <=20% of infusions after cutoff; PM group: >20% after cutoff	NR	NR	NR	NR
Nelson 2022 [48]	advanced solid tumors	anti-PD-1, anti-PD-L1, or dual anti PD-1/CTLA-4	4441	every 2 h from 8:00 to 20:00, and ‘overnight’ (20:00 to 8:00)	NR	NR	NR	NR	NR
Ortego 2022 [49]	metastatic urothelial cancer	anti-PD-1 or anti-PD-L1	92	16:30	AM group: <20% of infusions after cutoff; PM group: >=20% after cutoff	NR	NR	NR	NR
Pascale 2024 [50]	advanced hepatocellular carcinoma	Atezo	131	13:00	AM group: >=1 of the first two infusions before cutoff; PM group: first two infusions after cutoff	NR	NR	NR	NR
Rodriguez 2023 [51]	advanced or metastatic NSCLC	Pembro	276	16:30	AM group: first infusion before cutoff; PM group: first infusion after cutoff	NR	NR	NR	NR
Sun 2024 [52]	advanced cancer	Camre, Tisle	174 total; 109, camrelizumab cohort; 65, tislelizumab cohort	16:00	AM group: <1 infusion after cutoff; PM group: >=1 infusion after cutoff	any grade toxicity: camrelizumab cohort, AM group = 99%, PM group = 93%; tislelizumab cohort, AM group = 97%, PM group = 100%; grade 3 toxicity: camrelizumab cohort, AM group = 6%, PM group = 7%; tislelizumab cohort, AM group = NR, PM group = 13%; (any grade) anemia, 71% (camrelizumab) and 79% (tislelizumab); thrombocytopenia, 54% (camrelizumab) and 59% (tislelizumab); lymphopenia, 44% (camrelizumab and tislelizumab)	NR	NR	NR
van Rensburg 2022 [53]	advanced solid tumors	Pembro	106	12:00, 15:06, 15:11, or 16:30	AM groups: first infusion before 12:00 or 15:06, >=50% of infusions before 12:00 or 15:11, <20% of infusions after 16:30; PM groups: first infusion after 12:00 or 15:06, >=50% of infusions after 12:00 or 5:11, >=20% of infusions after 16:30	any grade ≥ 2; frequency of types NR	frequency of any grade ≥ 2 toxicity	no difference	NR
Vilalta 2021a [54]	NSCLC	anti-PD-1	197	12:00	AM group: >=1 of the first 4 infusions before cutoff; PM group: all 4 first infusions after cutoff	NR	NR	NR	NR
Vilalta 2021b [55]	NSCLC	anti-PD-1	105	12:00	AM group: >=1 of the first 4 infusions before cutoff; PM group: all 4 first infusions after cutoff	NR	NR	NR	NR

Landré 2024: Note that not all proportions were applied to each time-of-day cutoff. Abbreviations: NSCLC—non-small-cell lung cancer, RCC—renal cell carcinoma, HNSCC—head and neck squamous cell carcinoma, Atezo—Atezolizumab, Durva—Durvalumab, Nivo—Nivolumab, Pembro—Pembrolizumab, Ipi—Ipilimumab, Camre—Camrelizumab, Tisle—Tislelizumab, TOD—time of day, NR—not reported.
